# The initial experience of MRI-guided precision prone breast irradiation with daily adaptive planning in treating early stage breast cancer patients

**DOI:** 10.3389/fonc.2022.1048512

**Published:** 2022-11-23

**Authors:** John Ng, Ryan Pennell, Silvia C. Formenti

**Affiliations:** Department of Radiation Oncology, Weill Cornell Medicine, New York, NY, United States

**Keywords:** breast radiation therapy, partial breast irradiation, magnetic resonance image guided radiation therapy, precision radiation oncology, image guided adaptive radiotherapy

## Abstract

**Background:**

A major challenge in breast radiotherapy is accurately targeting the surgical cavity volume. Application of the emerging MRI-guided radiotherapy (MRgRT) technique in breast radiotherapy may enable more accurate targeting and potentially reduce side effects associated with treatment.

**Purpose:**

To study the feasibility of delivering MRI-guided partial breast radiotherapy or Precision Prone Irradiation (PPI) to treat DCIS and early stage breast cancer patients.

**Materials and methods:**

Eleven patients with diagnosed DCIS or early stage breast cancer treated with lumpectomy underwent CT-based and MRI-based simulations and treatment planning in the prone position. MRI-guided radiotherapy was utilized to deliver partial breast irradiation. A customized adaptive plan was created for each delivered radiotherapy fraction and the cumulative doses to the target volumes and nearby organs at risk were determined. The CT-based and the MRI-guided radiotherapy plans were compared with respect to target volumes, target volume coverage, and dose to nearby organs.

**Results:**

All patients receiving PPI successfully completed their treatments as planned. Clinical target volume (CTV) and planning target volume (PTV) dose coverage and organs-at-risk (OAR) dose constraints were met in all fractions planned and delivered and the MRI-guided clinical target volumes were smaller when compared to those of the CT-based partial breast radiotherapy plans for these eleven patients.

**Conclusions:**

MRI-guided partial breast radiotherapy as a breast radiotherapy technology is feasible and is a potential high clinical impact application of MRgRT. PPI has the potential to improve the therapeutic index of breast radiotherapy by more accurately delivering radiation dose to the cavity target and decreasing toxicities associated with radiation to the surrounding normal tissues. Prospective clinical data and further technical refinements of this novel technology may broaden its clinical implementation.

## Introduction

Patients with ductal carcinoma *in situ* or early stage breast cancer account for approximately 64% of newly diagnosed breast cancer patients (1). For early stage breast cancer patients, adjuvant radiotherapy after breast conserving surgery is the most commonly utilized treatment approach (49%) (2). Recent advances in breast radiotherapy have enabled shorter treatment courses, smaller radiation fields, prone positioning, and less side effects associated with breast cancer treatment (3–10).

Magnetic Resonance-guided Radiotherapy (MRgRT) for breast cancer treatment is a potential high impact application of an emerging radiotherapy technology (11, 12). Potential advantages of radiotherapy utilizing on-table Magnetic Resonance Imaging (MRI) include enhanced visualization of the surgical cavity after lumpectomy and real time visualization and motion management of the cavity and nearby organs at risk (OARs). Furthermore, adaptive planning with MRgRT enables customized daily treatment planning to account for day-to-day anatomical variation. Finally, prone positioning during breast irradiation leads to decreased lung and heart radiation doses during treatment (3). By combining MRgRT and adaptive planning with prone positioning, a technique we call precision prone irradiation (PPI), we can improve the therapeutic index of breast radiotherapy by more accurately delivering radiation dose to the cavity target and decreasing toxicities associated with radiation to the surrounding normal tissues.

In this study, eleven patients with either early stage breast cancer or ductal carcinoma *in situ* (DCIS) were treated with PPI utilizing prone breast MRgRT and adaptive planning as their adjuvant breast radiotherapy treatment. To our knowledge, this is the first reported patient experience with this technology to date.

## Materials and methods

Eleven patients with diagnosed DCIS or early stage breast cancer who underwent lumpectomy with negative margins underwent CT and MRI-based simulations and treatment planning. The patients consented for and underwent adjuvant breast radiotherapy treatments at New York Presbyterian Hospital – Weill Cornell Medicine with Eastern Cooperative Oncology Group Performance status (13) of 0-1 and histologically confirmed breast cancer between March 2021 and July 2021. This retrospective series included the first eleven consecutive patients who received prone MRI-guided breast radiotherapy and who are undergoing regular oncological follow-up examinations in our clinic. The characteristics of these eleven patients are shown in **Table 1**.

**Table 1:** Patients Characteristics – (NA: Not applicable, ER: Estrogen Receptor, PR: Progesterone Receptor).

Patient Number	Age	Right/Left Breast	TNM Anatomic Pathology Stage	ER/PR/Her-2 Status
1	60	Right	T1b N0	+/+/-
2	74	Right	T1c N0	+/+/-
3	71	Left	T1b N0	+/+/-
4	71	Right	T1c N0	+/+/-
5	65	Left	Tis	+/+/NA
6	72	Left	T1a N0	+/+/-
7	72	Right	T1b N0	+/-/-
8	63	Left	T1c N0	+/+/-
9	58	Left	T1a N0	+/+/-
10	68	Right	T1b N0	+/+/-
11	65	Left	T1b N0	+/+/-

Before simulation, all patients were screened for MRI compatibility. All patients were treated using a 0.35 T hybrid MR Linac system (MRIdian, ViewRay Inc., Mountain View, CA). All patients were planned in the prone breast treatment position within one to three months of surgical resection in accordance with previous published radiotherapy protocols (14, 15). The patients underwent MR simulation on the 0.35 MRIdian system using a balanced steady-state free progression (TrueFISP) imaging sequence. Planning CT using the same patient positioning was performed to acquire the electron density information for treatment planning. The two simulation image sets were fused and target contour delineation was performed on the CT and MRI simulation scans.

On each day of radiation treatment delivery, the surgical bed clinical target volume (CTV) and organs at risk (OAR) (left anterior descending artery, heart, lungs, and breast) were delineated on the MRI simulation scans. The CTV was isotropically expanded by 1.0 to 1.5 cm margins to define the planning target volume (PTV). The dose prescription was 30 Gy in 5 fractions given every other day. The planning and OAR constraints were based on previous published radiotherapy protocols (14, 15). Treatment planning was performed with Varian Eclipse and MRIdian treatment planning software (MC dose calculation algorithm) and treatment delivery consisted of a step and shoot IMRT technique. The linear accelerator radiation delivery utilizes a 6MV flattening free filter.

**Figure 1:**
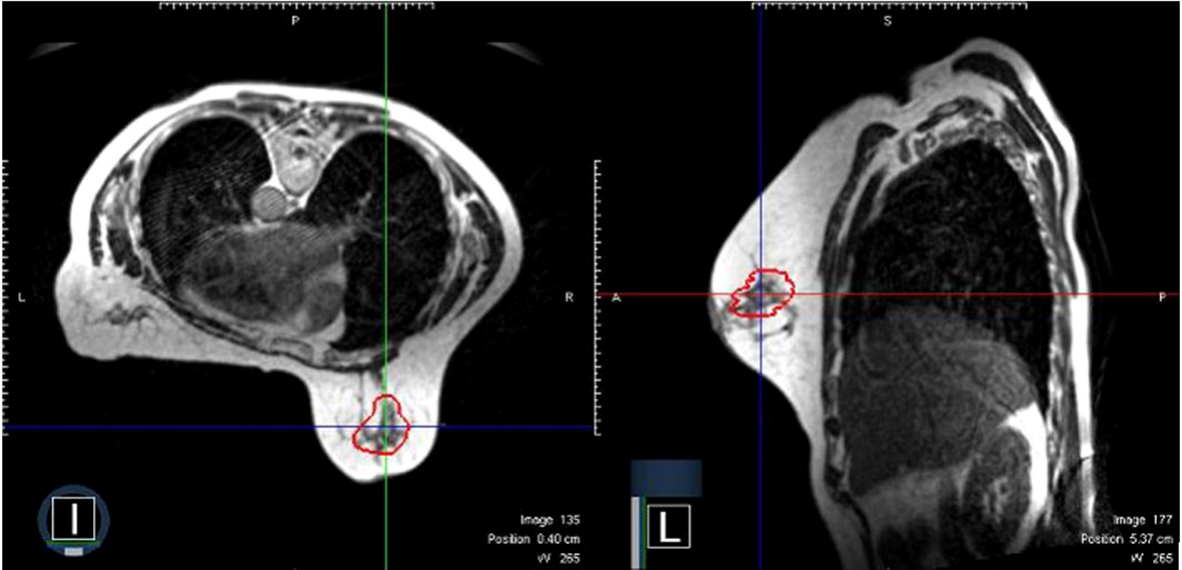
A representative surgical cavity target volume seen from an MRI image acquired on-table on the day of radiation treatment. The MRI scans in both the axial and sagittal planes are shown. The surgical cavity is outlined in red.

All patients received partial breast radiotherapy delivered in the prone treatment position with tangential Intensity Modulated Radiation Therapy (IMRT) fields. Each fractional treatment used an on-table adaptive planning workflow, where a new treatment plan was optimized after same day re-contouring of the anatomy from MRI imaging acquired on that day. As each fraction was adaptively planned, there were no imaging shifts used for patient setup. During the radiation delivery, continuous real-time 2D-cine-MRI was used to control for organ and target motion. A target structure was defined in the sagittal view of the volumetric MRI and a surrounding gating boundary contour was created by adding a tracking margin of 3 mm. An example of a surgical cavity target volume delineated from an MRI image acquired on-table on the day of radiation treatment is shown in **Figure 1**.

All standard radiation treatment plans and dosimetry parameters as described above were stored and analyzed on the Varian Eclipse and MRIdian treatment planning software system. Toxicity was assessed and reported according to Common Terminology Criteria for Adverse Events (CTCAE) v5 and recorded on the hospital’s electronic medical record system.

## Results

All patients were able to complete their treatments as planned. Each radiotherapy fraction was adapted to the image acquired on the day of treatment and each adaptive plan successfully met pre-specified dose constraints.

For the delivered treatments based on the daily MRI images, the mean CTV size was 20.2 cc (range: 11.6-33.9 cc) and the mean PTV volume was 78.9 cc (56.8-124.6 cc). For the adapted fractional planning, the mean CTV size was 21.8 cc (3.9-37.4cc). The median LAD maximum dose was 44.5 cGy and the median mean heart dose was 15.5 cGy. The median percentage of the lung getting above 20 Gy dose was 0%. The median % of breast volume getting 100% and 50% of the prescription dose was 15.0% and 34.7% respectively. The average treatment time for an adapted fraction delivery was 38 minutes. Other pertinent dosimetry and treatment characteristics are summarized in **Table 2**.

**Table 2:** Pertinent dosimetry characteristics of the eleven patient treatment plans.

Patient Number	Right/Left	CTV Volume (cc)	PTV Eval Volume (cc)	Mean Heart (cGy)	LAD Max (cGy)	Ipsilateral V20 Dose (%)	Average of total treatment time (min)
1	R	32.1	124.6	13	15	0	34.7
2	R	26.7	118.6	13	17	0	36.4
3	L	12.3	52.5	14	59	0	35.8
4	R	10.9	64.2	13	19	0	38.6
5	L	12.6	83	25	146	0	37.2
6	L	14.8	72.9	20	85	0	34.4
7	R	18.1	85	11	18	0	37.6
8	L	33.9	115.3	22	79	0.01	38.4
9	L	11.6	56.8	31	67	0	39.2
10	R	14.8	72.7	17	30	0	44.3
11	L	2.1	22.4	11	41	0	37.2

(V20: Volume of lung tissue receiving 20 Gy or more, cGy: Centigray, LADMax: Maximum dose to the left anterior descending artery, CTV: Clinical Target Volume, cc: cubic centimeters, PTV Eval: Planning Target Volume Evaluation).

Alternative treatment plans were developed based on the conventional CT simulation scans for the eleven patients. In comparison to the MRI plans, the mean CTV size based on alternative CT plan was greater, measuring 73.7 cc (range: 10.2–147.3 cc). The median difference in CTV volumes between CT-based and MRI-based planning was 45.2 cc (range 7.3 cc to 115.2 cc).

The median follow up for all eleven patients treated was 6 months. At time of this report, none of the patients have reported any Grade 2 or higher toxicities. All eleven patients tolerated treatment well with all patients reporting Grade 1 erythema of the skin which had resolved by their first follow up at 4 weeks post-radiotherapy. None of the patients reported any Grade 2 or higher fatigue or any additional adverse side effects.

## Discussion

We report on our initial clinical experience of MRI-guided Radiation Therapy (MRgRT) with daily adaptive treatment planning for patients receiving breast radiotherapy in the prone position with a MRI-Linac radiotherapy system. As far as we are aware, this is the first reported clinical experience of breast radiotherapy delivered using the PPI technique.

In early-stage breast cancer management, there is significant momentum towards de-escalating intensity of treatment. Local recurrence rates in early stage disease have decreased over time and are reported to be less than 5% over 10 years of follow up in recent clinical trials (5, 14, 15). Nevertheless, fear of recurrence after treatment and long-term toxicities associated with treatment remain primary issues in survivorship, and local and distal breast cancer recurrence risk remains a concern even 20 years after treatment for early stage breast cancer patients (16).

De-escalation in breast radiotherapy has emphasized treating smaller target volumes and prioritizing avoiding nearby heart and lungs. A current standard breast radiotherapy modality involves external beam partial breast radiotherapy with CT-based image guidance. Several large Phase 3 trials have demonstrated good efficacy and toxicity of external beam partial radiotherapy when compared with whole breast radiotherapy (5, 15). In this report, we present our initial patient experience of precision prone irradiation (PPI) with MRI-guided prone breast adaptive breast radiotherapy for early stage breast cancer treatment. Our preliminary comparative results indicate that there may be a potential to decrease CTV and PTV volumes based on MRI guided planning.

Image-guided radiotherapy technologies are improving in sophistication and versatility to enhance the ability to target the at-risk regions and avoid healthy tissues during radiation treatments. The capability of on-table MRI guidance, adaptive planning, and delivery with the prone technique has several particular promise advantages to expand the therapeutic index of breast radiotherapy.

First, MRI enables the ability to delineate soft tissue and surgical cavities to a great resolution than CT-based imaging. This superior visualization by MRI guidance can allow more accurate surgical cavity and smaller target volume delineation as suggested from the results of our study. In addition, MRI guidance potentially allows CTV and PTV margins to be reduced from current standards due to better cavity determination and less setup uncertainty. Intra-fractional MR-guided imaging and decreased chest wall excursion with the prone setup furthers accounts for organ motion and can decrease targeting uncertainty (17). With potentially smaller radiation treatment volumes, this technique may decrease skin toxicities associated with breast radiotherapy with current published estimates of grade 2+ NCI CTCAE toxicity to be 15-20% with partial breast radiotherapy and 70-80% with whole breast radiotherapy (18).

The versatility of daily adaptive imaging, contouring, planning, and radiation delivery process can help mitigate the challenges of daily setup uncertainties for breast radiotherapy. The surgical cavity can be difficult to delineate without surgical clip or marker placement within the surgical bed, a practice that varies amongst surgeons. When surgical clips are placed to mark the cavity, they can migrate from their initial position within the breast over time. It is known that the surgical cavity can change through a course of breast radiotherapy (19). Furthermore, the positional setup and the position of the breast tissue can be highly variable during daily setups. Significant changes to the breast surface and topology can occur during the course of radiation treatment (20).

Similar to the breast target, nearby organs at risk can also change in relative position with daily treatments. Cardiac positioning can vary considerably relative to the bony anatomy and other anatomic landmarks used for patient positioning (21). Even with prone positioning, the left anterior descending coronary artery can receive significant radiation dose if daily imaging guidance is not utilized (13). For those patients where positional setup uncertainty is significant, it is recommended that more frequent image-guidance be utilized during breast radiotherapy, such as daily cone-beam CTs (22). PPI enables greater flexibility and potentially more accurate treatment delivery.

Incorporation of PPI and utilizing MR image guidance for breast radiation treatment planning enables minimization or potentially elimination of CT scanning for patients in the simulation and treatment delivery process. CT-based treatment planning often requires daily cone beam CT imaging for setup verification. For patients with high aversion to CT scans or tattoo marking, MRgRT offers the opportunity to potentially bypass the CT simulation, permanent skin tattooing, or regular cone beam CT image verifications which have become standard procedures in the contemporary breast radiotherapy delivery process.

At this stage of the PPI technology development, there are still significant limitations to broad clinical utilization in routine breast radiotherapy. MRI guidance, motion management, and daily adaptive planning are resource intensive and requires the presence of clinicians, dosimetrists, physicists, and therapists at each treatment. The time required for each delivered PPI fraction is longer than that for standard CT-imaged guided breast radiotherapy. The potential promise of smaller radiation treatment volumes through better target delineation and less target uncertainty and decreased toxicities would need to be validated through a prospective clinical trial.

With our reported experience, we hope to demonstrate potential advantages of PPI over conventional CT-based breast radiotherapy. The significance of these advantages will have to be established through prospective clinical trials. As further advances occur in this rapidly emerging field, we expect these clinical advantages and more therapeutic benefits to be realized from this versatile technique.

## Data availability statement

The original contributions presented in the study are included in the article. Further inquiries can be directed to the corresponding author.

## Ethics statement

The studies involving human participants were reviewed and approved by Weill Cornell Medicine Institutional Review Board. Written informed consent for participation was not required for this study in accordance with the national legislation and the institutional requirements.

## Author contributions

All authors listed have made a substantial, direct, and intellectual contribution to the work and approved it for publication.

## Conflict of interest

The authors declare that the research was conducted in the absence of any commercial or financial relationships that could be construed as a potential conflict of interest.

## Publisher’s note

All claims expressed in this article are solely those of the authors and do not necessarily represent those of their affiliated organizations, or those of the publisher, the editors and the reviewers. Any product that may be evaluated in this article, or claim that may be made by its manufacturer, is not guaranteed or endorsed by the publisher.

## Abbreviations

CTV, Clinical Target Volume; DCIS, Ductal Carcinoma in Situ; IMRT, Intensity Modulated Radiation Therapy; MRgRT, Magnetic Resonance guided Radiation Therapy; OAR, Organs at Risk; PBI, Partial Breast Irradiation; PPI, Precision Prone Irradiation; PTV, Planning Target Volume.
